# All-silicon terahertz metasurfaces for multi-focus multiplexed polarization generation

**DOI:** 10.1016/j.isci.2025.114458

**Published:** 2025-12-16

**Authors:** Susu Hu, Yongzheng Lu, Tianchen Tang, Li Wei, Bo Dai, Songlin Zhuang, Dawei Zhang

**Affiliations:** 1 Engineering Research Center of Optical Instrument and System, The Ministry of Education, Shanghai Key Laboratory of Modern Optical System, University of Shanghai for Science and Technology, Shanghai 200093, China; 2 School of Photoelectric Engineering, Changzhou Institute of Technology, Changzhou 213032, China

**Keywords:** physics, optics, applied sciences

## Abstract

Polarization multiplexing enhances communication capacity but faces material limitations requiring complex device cascading. This paper proposes a strategy for generating multiple polarization states by combining geometric phase and propagation phase in spatially interleaved meta-atoms. This strategy enables the simultaneous generation of scalar (including linearly polarized, circularly polarized) and vector beams (VBs) in the transmitted wave, along with precise wavefront shaping capabilities for these distinct polarization states. Two all-silicon terahertz metasurfaces are designed that are capable of independently manipulating both the phase and amplitude of different polarization states in transmission. The first metasurface achieves focusing with multi-polarization states under left-handed circularly polarized (LCP) illumination, while the second realizes longitudinal focusing of vortex beams exhibiting multiple polarization states under LCP excitation, with each vortex beam carrying unique topological charges. This approach paves new avenues for advanced terahertz polarization manipulation, offering significant potential for next-generation communication and secure information transmission.

## Highlights

• Interleaved meta-atoms to generate multiple polarization states and vector beams
• All-silicon metasurfaces enable independent control of phase and amplitude
• Longitudinal multi-polarization vortex beams with unique topological charges under LCP

## Graphical abstract

**Figure:**
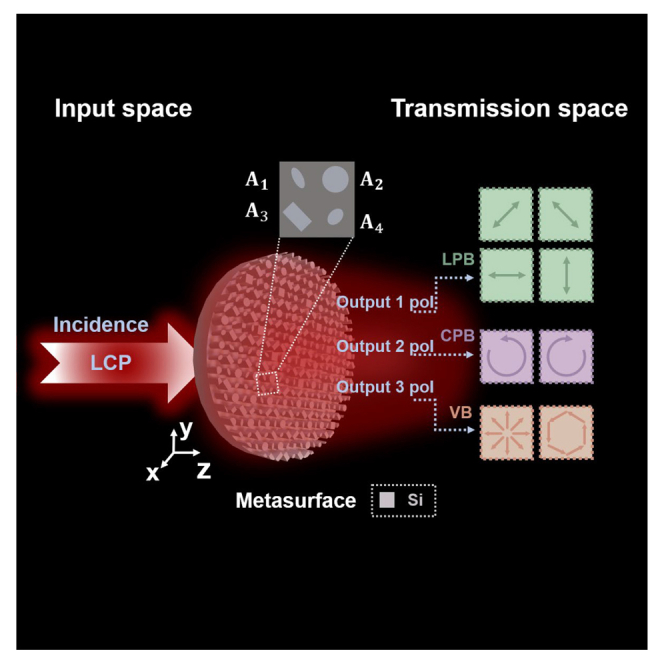

## Introduction

Traditional optical components rely on continuous accumulation of phase propagation within materials to achieve wavefront manipulation. In contrast, metasurfaces utilize the unique control mechanism of abrupt phase shifts, enabling arbitrary wavefront control on an ultra-compact platform, thus enhancing system miniaturization and integration.^1^^,^^2^^,^^3^ By precisely engineering the geometric configuration, dimensional parameters, spatial orientation, and array arrangement of constituent units, metasurfaces introduce controllable phase gradient distributions at interfaces, thereby achieving customized wavefront manipulation.^4^^,^^5^^,^^6^^,^^7^^,^^8^^,^^9^ Beyond phase modulation, these artificial metasurfaces demonstrate multidimensional control over key optical parameters including amplitude,^10^^,^^11^^,^^12^ frequency,^13^^,^^14^^,^^15^ and polarization state.^16^^,^^17^^,^^18^^,^^19^^,^^20^^,^^21^ This versatile capability has facilitated widespread applications in areas such as perfect absorbers,^22^^,^^23^^,^^24^ structural color generation,^25^^,^^26^ color holography,^27^^,^^28^^,^^29^^,^^30^^,^^31^^,^^32^ achromatic optical elements,^33^^,^^34^ and nonlinear optics.^35^^,^^36^^,^^37^ It is worth noting that the metasurfaces have exceptional capacity for polarization manipulation at subwavelength scales.^38^^,^^39^^,^^40^^,^^41^^,^^42^^,^^43^^,^^44^

The integration of Pancharatnam-Berry (PB) phase and propagation phase enables multifunctional wavefront control under circularly polarized (CP) illumination, achieving efficient polarization conversion and beam steering.^45^ However, the generation and simultaneous manipulation of complex multiple polarization states remain challenging due to the intrinsic coupling between phase and polarization modulation in conventional designs. Recent studies have primarily focused on decoupling polarization and phase control through strategies such as interleaved nanostructures or multiplexed meta-atoms.^46^ For instance, a new metasurface paradigm was proposed that can completely manipulate the amplitudes and phase of two spin components based on the interference effect. Two five-channel meta-holograms for imaging and information encryption were designed for demonstration.^47^ With the deepening of research, independent manipulation of orthogonal polarization and simultaneous multifunctional wavefront shaping was realized based on spin decoupling.^48^ Then, a generalized framework for independent phase control and arbitrary energy distribution of different polarization channels was proposed. And tri-polarization channel wavefront control for the arbitrary polarization state was experimentally demonstrated.^49^ Despite these advances made, existing methods primarily generate simplified polarization distributions (e.g., linear or circular polarization states) and lack the flexibility to create high-efficiency spatially varying multiple polarization states. This limitation hinders applications in advanced polarization optics, including vector beams (VBs) generation, vortex beam creation, multi-polarization-encoded imaging, and quantum information processing.

Here, we propose a framework with an all-dielectric metasurface integrating PB phase with propagation phase to address these challenges. The designed meta-molecule structure enables spatially varying transitions from single polarization states to multi-polarization states, including orthogonal CP states and orthogonal linearly polarized (LP) states. By incorporating amplitude-encoded phase modulation into the functional metasurfaces design, our approach achieves precise and independent control over multiple polarization transmission channels. Furthermore, the proposed strategy is not only capable of generating in-plane complex multi-polarization states in two-dimensional (2D) geometries but also extends to longitudinal manipulation for creating complex multi-polarized vortex beams. This work advances polarization optics by establishing a unified framework for generating multiple multi-polarization states, with potential applications in optical encryption, super-resolution imaging, and high-capacity communication systems.

## Design and method

### Scheme of multiple polarization states generation

**Figure 1:**
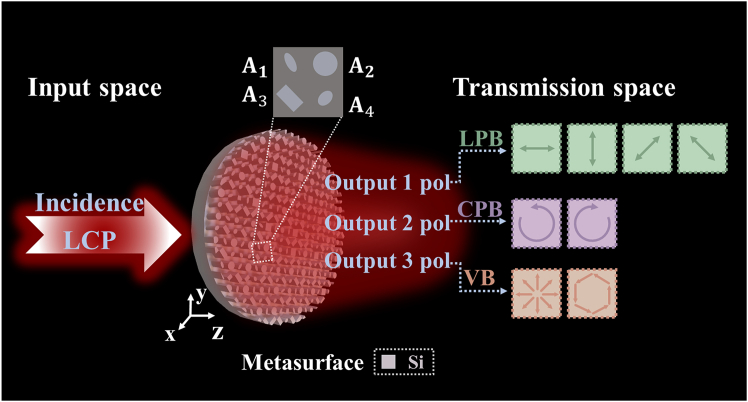
Schematic of multiple polarization states generation Spatially interleaved meta-atoms constitute meta-molecules. By introducing phase information and amplitude encoding, the metasurface can generate various linearly polarized beams (LPBs), circularly polarized beams (CPBs), and VB under LCP incident wave.

The scheme of an all-silicon terahertz metasurface composed of spatially interleaved meta-atoms is proposed to induce the generation of co-polarization, cross-polarization, multiple linear polarization, and radial/angular polarization simultaneously. As shown in Figure 1, the meta-molecules constituting the metasurface are composed of four types of meta-atoms (A_1_, A_2_, A_3_, and A_4_). Under illumination of left-handed circularly polarized (LCP) wave, the anisotropic meta-atoms A_1_ manipulate the cross-polarized wave using the PB phase, which is introduced by adjusting the rotation angles of the atoms. The meta-atoms A_2_ introduce the propagation phase by adjusting the geometric parameters of the pillar to manipulate the incident co-polarized wave, aiming to induce the generation of co-polarization states in the transmitted wave. The meta-atoms A_3_ is rotated 45° counterclockwise to induce *y* linearly polarized (*y*-LP) waves, and A_4_ is rotated 45° clockwise to induce *x* linear polarized (*x*-LP) waves. Both A_3_ and A_4_ employ the propagation phase to manipulate the conversion of incident CP waves to LP waves. By incorporating essential phase profiles and amplitude information into the metasurface design, the phase of the four different polarized transmitted waves can be flexibly controlled. Consequently, beam interference can be achieved by superimposing two waves with specific phase differences at the same position, resulting in multiple polarization states in the transmitted wave. Ultimately, the beam interference in the transmitted wave can generate +45° linearly polarized (+45°-LP) and −45° linearly polarized (−45°-LP) beams, radially polarized vector beams (RPVB) and azimuthally polarized vector beams (APVB).

**Figure 2:**
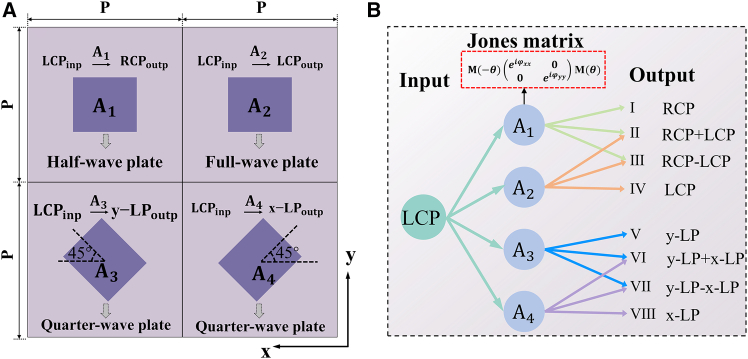
The generation principle of complex multi-polarization (A) Configuration schematic of the meta-molecule layout. Elements A_1_ and A_2_ need not be rotated in space, while A_3_ and A_4_ are rotated by ±45° in space along the *z* axis. A_1_ exhibits HWP characteristics, enabling the conversion of LCP beams to RCP beams. A_2_ demonstrates full-wave plate properties to manipulate the LCP component in transmitted waves. A_3_ and A_4_ function as quarter-wave plates, converting LCP beams into *x*-LP and *y*-LP components, respectively. (B) Schematic of beam interference by superimposing two waves.

Figure 2 illustrates the generation principle of complex multi-polarization. Figure 2A shows the meta-molecule constituting the metasurface, which comprises four types of meta-atoms with distinct physical properties. Meta-atoms A_1_ functions as a half-wave plate (HWP) with a phase retardation of Δ*φ* = *φ*_*xx*_ – *φ*_*yy*_ = *π* between its fast and slow axes. When the rotation angle is 0°, its corresponding Jones matrix can be expressed as (see Section S1 in the supplemental information for the detailed derivation):

(Equation 1) $$J_{A_{1}}=\left[\begin{array}{cc} e^{i\varphi_{xx}} & 0\\ 0 & -e^{i\varphi_{xx}} \end{array}\right]$$

For LCP incident light, the output light is

(Equation 2) $$E_{out1}=J_{A_{1}}\times E_{in}=\left[\begin{array}{cc} e^{i\varphi_{xx}} & 0\\ 0 & -e^{i\varphi_{xx}} \end{array}\right]\times \left[\begin{array}{l} 1\\ -i \end{array}\right]=e^{i\varphi_{xx}}\left[\begin{array}{l} 1\\ i \end{array}\right]$$

Meta-atoms A_2_, exhibits a phase retardation of Δ*φ* = *φ*_*xx*_ – *φ*_*yy*_ = 0 between its fast and slow axes, its corresponding Jones matrix is expressed as (see Section S1 in the supplemental information for the detailed derivation).

(Equation 3) $$J_{A_{2}}=\left[\begin{array}{cc} e^{i\varphi_{xx}} & 0\\ 0 & e^{i\varphi_{xx}} \end{array}\right]$$

resulting in an output light as,

(Equation 4) $$E_{out2}=J_{A_{2}}\times E_{in}=\left[\begin{array}{cc} e^{i\varphi_{xx}} & 0\\ 0 & e^{i\varphi_{xx}} \end{array}\right]\times \left[\begin{array}{l} 1\\ -i \end{array}\right]=e^{i\varphi_{xx}}\left[\begin{array}{l} 1\\ -i \end{array}\right]$$

Meta-atoms A_3_ and A_4_ exhibit the characteristics of quarter-wave plates, with phase retardation between their fast and slow axes being Δ*φ* = *φ*_*xx*_ – *φ*_*yy*_ = *π*/2. When A_3_ and A_4_ are rotated by angles of *π*/4 and −*π*/4, respectively, they follow that (see Section S1 in the supplemental information for the detailed derivation).

(Equation 5) $$J_{A_{3}}=\frac{\sqrt{2}}{2}e^{i\varphi_{xx}}\left[\begin{array}{cc} e^{i\frac{\pi }{4}} & e^{-i\frac{\pi }{4}}\\ e^{-i\frac{\pi }{4}} & e^{i\frac{\pi }{4}} \end{array}\right]$$

(Equation 6) $$E_{out3}=J_{A_{3}}\times E_{in}=\sqrt{2}e^{i\left(\varphi_{xx}-\frac{\pi }{4}\right)}\left[\begin{array}{l} 0\\ 1 \end{array}\right]$$

(Equation 7) $$J_{A_{4}}=\frac{\sqrt{2}}{2}e^{i\varphi_{xx}}\left[\begin{array}{cc} e^{i\frac{\pi }{4}} & -e^{-i\frac{\pi }{4}}\\ -e^{-i\frac{\pi }{4}} & e^{i\frac{\pi }{4}} \end{array}\right]$$

(Equation 8) $$E_{out4}=J_{A_{4}}\times E_{in}=\sqrt{2}e^{i\left(\varphi_{xx}+\frac{\pi }{4}\right)}\left[\begin{array}{l} 1\\ 0 \end{array}\right]$$

Then, the secondary energy redistribution among the four polarization states in transmitted waves enables superposition of spatially diverse polarization states to generate multiple derived polarization states, as illustrated in Figure 2B. Superposition of right-handed circularly polarized (RCP) and LCP vortex waves with equal amplitudes and identical or opposite initial phases generates RPVB or APVB (see Section S1 in the supplemental information for specific analysis). The superposition of *x*-LP and *y*-LP components with identical amplitudes and phases generates +45°-LP states. When introducing a phase difference of *π* between *x*-LP and *y*-LP components, beam interference by superimposing two waves will become −45°-LP states (see Section S1 in the supplemental information for the detailed derivation).

### Meta-atoms design and verification

**Figure 3:**
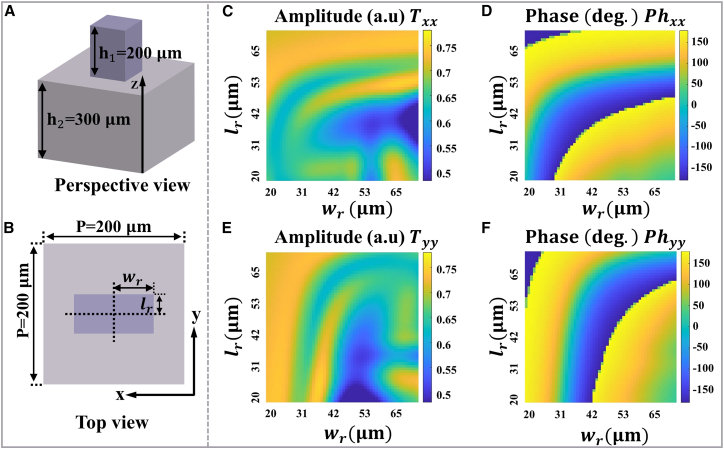
Rectangular column meta-atoms (A) Perspective view. (B) Top view. (C–F) The transmission coefficients (*T*_*xx*_, *T*_*yy*_) and phase shifts (*Ph*_*xx*_, *Ph*_*yy*_) of rectangular meta-atoms under *x*- and *y*-polarized illumination at 1.0 THz, as functions of geometric parameters (*w*_*r*_, *l*_*r*_).

**Figure 4:**
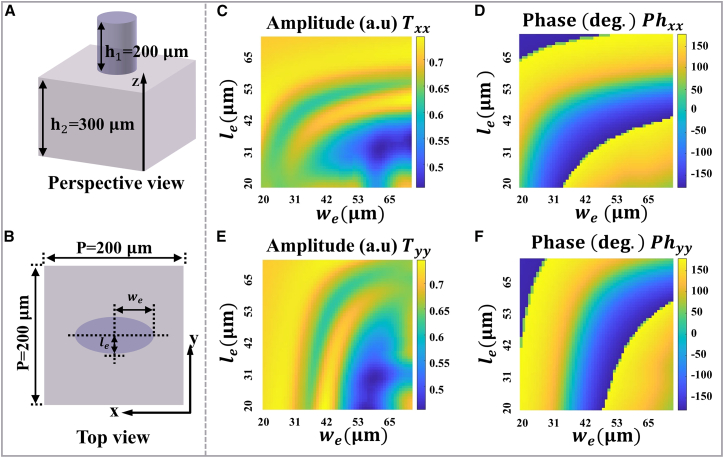
Elliptical cylinder meta-atoms (A) Perspective view. (B) Top view. (C–F) The transmission coefficients (*T*_*xx*_, *T*_*yy*_) and phase shifts (*Ph*_*xx*_, *Ph*_*yy*_) of elliptical meta-atoms under *x*- and *y*-polarized illumination at 1.0 THz, as functions of geometric parameters (*w*_*e*_, *l*_*e*_).

Parameter scan simulations were performed in CST Microwave Studio, employing periodic boundaries along the *x*- and *y*-directions and open boundary along the *z*-direction. It is worth noting that our primary design requirement for the three meta-atoms types (A_1_–A_3_) was to obtain continuous 0–2*π* phase coverage while maintaining the required transmission. To achieve that, we performed parameter scan over two kinds of unit-cell geometries. In those scans, we found that a library composed solely of one geometry library (only elliptical pillars or only rectangular pillars) could not provide a complete 0–2*π* phase sweep within the chosen parameter ranges. By combining different geometry libraries, we were able to populate complementary regions of phase space and thereby achieve continuous phase coverage together with high transmission. The basic units are illustrated in Figures 3 and 4 (see Tables S1–S3 in the supplemental information for the detailed geometric parameters). The rectangular column and the elliptic cylinder serve as all-silicon meta-atoms, leveraging silicon’s high permittivity (*ε* = 11.9) in the terahertz band to achieve effective optical field confinement while maintaining sufficiently low crosstalk between adjacent elements. This characteristic enables each meta-atom to approximate an independent Jones matrix pixel, thereby facilitating high-precision wavefront control. The lattice period of the unit cells is *p* = 200 μm, with a substrate height of *h*_2_ = 300 μm and a pillar height of *h*_1_ = 200 μm. Figures 3A, 3B, 4A, and 4B, respectively, show the perspective and top views of rectangular column and elliptical cylinder meta-atoms. The corresponding transmission coefficients (*T*_*xx*_, *T*_*yy*_) and phase shifts (*Ph*_*xx*_, *Ph*_*yy*_) under *x*/*y*-polarized illumination at 1.0 THz are presented in Figures 3C–3F and 4C–4F), as functions of their geometric parameters (*w*_*r*_, *l*_*r*_) and (*w*_*e*_, *l*_*e*_), respectively.

**Figure 5:**
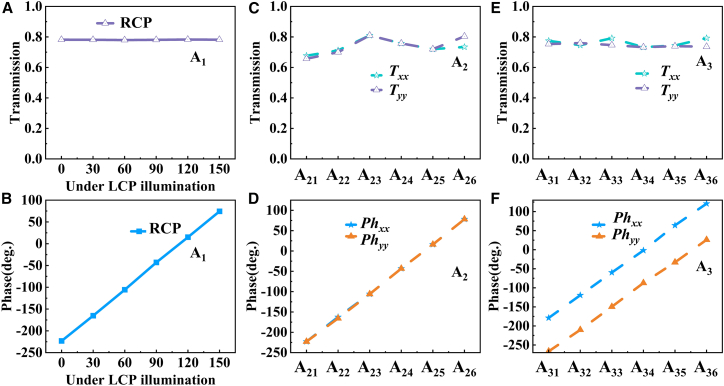
The selected three types of meta-atoms (A) Transmission amplitudes. (B) corresponding phase distributions for A_1_-type meta-atoms under LCP illumination, as functions of spatial rotation angle. (C) Transmission amplitudes. (D) Phase distributions of A_2_-type meta-atoms under *x*- and *y*-LP illumination. (E and F) Transmission amplitudes and (F) phase profiles of A_3_-type meta-atoms under *x*- and *y*-linear polarized illumination.

To effectively manipulate the wavefront, the phase shifts must span the full 2*π* range. Based on parameter scanning results, we selected three types of meta-atoms that fulfill the design requirements, as illustrated in Figure 5. Unlike propagation phase, which typically relies on laborious structural parameter optimization for CP light, numerous studies have demonstrated that PB phase modulation can be achieved simply by rotating meta-atoms. Therefore, for the A_1_ configuration, we implemented PB phase-based wavefront manipulation by selecting met a-atoms whose initial phases closely matched those of A_21_ to establish coherent superposition conditions.

As shown in Figures 5A and 5B, a full 2*π*-phase coverage can be obtained by rotating the A_1_ clockwise in the space with each rotation of *π*/6 under LCP illumination. Remarkably, the amplitude of the RCP transmitted wave remains stable at approximately 0.8. Figures 5C and 5D display the transmission amplitudes and phase distributions of six A_2_-type meta-atoms under *x*- and *y*-polarized illumination. The transmission amplitudes maintain nearly identical values (∼0.76), while the phase distributions of A_21_–A_26_ span the full 0–2*π* range. Figures 5E and 5F present the performance of six A_3_-type meta-atoms. These selected units demonstrate nearly identical transmission amplitudes (∼0.8) under *x*- and *y*-polarized illumination, with the phase distribution of A_31_–A_36_ also covering the complete 0–2*π* range.

## Results and discussion

### Generation of multiple polarization states in a single plane

**Figure 6:**
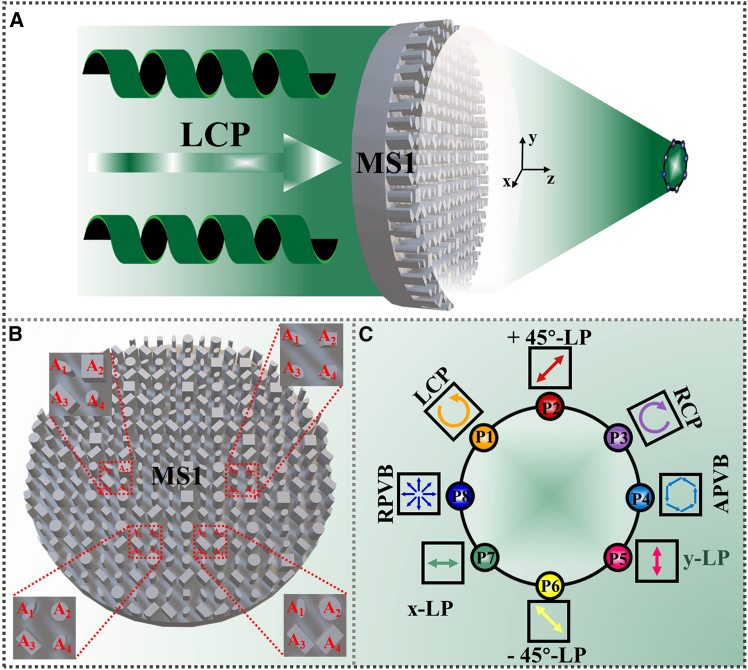
The first designed metasurface 8P1P-MS (A) Schematic illustration of complex multi-polarization states generated under LCP illumination. (B) Top view of 8P1P-MS. (C) Schematic of multiple polarization states distribution.

To validate the proposed strategy, we first designed metasurface, named as eight polarization states on one plane (8P1P-MS), capable of generating multiple states in the focal plane under LCP illumination (Figure 6A). As depicted in the top-view schematic (Figure 6B), 8P1P-MS consists of molecules containing four functionally distinct all-silicon nanopillars (A_1_–A_4_). Through spatial allocation of transmitted wave polarization components and amplitude encoding, energy equalization at interference points is achieved. This enables simultaneous realization of multiple polarization states in the focal plane, including LCP, RCP, x-LP, y-LP, ±45°-LP, RPVB, and APVB, as shown in Figure 6C.

In 8P1P-MS design, amplitude encoding is introduced to focus the transmitted waves in accordance with a specified energy distribution. Based on the method of complex amplitude modulation, the amplitude information of the complex field was encoded into LCP and RCP components, as formulated in Equations 9 and 10,

(Equation 9) $$\begin{array}{l} \varphi_{L1}=-\frac{2\pi }{\lambda }\left(\sqrt{{\left(x+1,400\right)}^{2}+y^{2}+f^{2}}-f\right)+\theta \\ \varphi_{L2}=-\frac{2\pi }{\lambda }\left(\sqrt{{\left(x+987\right)}^{2}+{\left(y-987\right)}^{2}+f^{2}}-f\right)\\ \varphi_{L3}=-\frac{2\pi }{\lambda }\left(\sqrt{{\left(x-1,400\right)}^{2}+y^{2}+f^{2}}-f\right)+\theta +\pi \\ E_{L}=l_{1}\;\exp\left(i\varphi_{L1}\right)+l_{2}\;\exp\left(i\varphi_{L2}\right)+l_{3}\;\exp\left(i\varphi_{L3}\right)\\ \varphi_{L}=abs\left(E_{L}\right)\times angle\left(E_{L}\right) \end{array}$$

(Equation 10) $$\begin{array}{l} \varphi_{R1}=-\frac{2\pi }{\lambda }\left(\sqrt{{\left(x+1,400\right)}^{2}+y^{2}+f^{2}}-f\right)-\theta \\ \varphi_{R2}=-\frac{2\pi }{\lambda }\left(\sqrt{{\left(x-987\right)}^{2}+{\left(y-987\right)}^{2}+f^{2}}-f\right)\\ \varphi_{R3}=-\frac{2\pi }{\lambda }\left(\sqrt{{\left(x-1,400\right)}^{2}+y^{2}+f^{2}}-f\right)-\theta \\ E_{R}=r_{1}\;\exp\left(i\varphi_{R1}\right)+r_{2}\;\exp\left(i\varphi_{R2}\right)+r_{3}\;\exp\left(i\varphi_{R3}\right)\\ \varphi_{R}=abs\left(E_{R}\right)\times angle\left(E_{R}\right) \end{array}$$

Here, the focal length *f* is designed as 4,000 μm, with the incident wavelength *λ* corresponding to a frequency of 1.0 THz. Under LCP illumination, the amplitude ratio of three foci of the generated LCP component is *l*_1_:*l*_2_:*l*_3_ = 1:2:1, as is the RCP component *r*_1_:*r*_2_:*r*_3_ = 1:2:1. Additionally, *φ*_*L*_ and *φ*_*R*_ represent the phase profiles of the LCP and RCP components in the output wave, respectively. *θ* = arctan(*y*/*x*) is the rotation azimuth with respective to the origin.

To obtain more polarization states, based on the method of complex amplitude modulation, we need to encode complex amplitude information into *x*-LP and y-LP components simultaneously, which can be described as Equations 11 and 12,

(Equation 11) $$\begin{array}{l} \varphi_{x1}=-\frac{2\pi }{\lambda }\left(\sqrt{x^{2}+{\left(y-1,400\right)}^{2}+f^{2}}-f\right)\\ \varphi_{x2}=-\frac{2\pi }{\lambda }\left(\sqrt{{\left(x+987\right)}^{2}+{\left(y+987\right)}^{2}+f^{2}}-f\right)\\ \varphi_{x3}=-\frac{2\pi }{\lambda }\left(\sqrt{x^{2}+{\left(y+1,400\right)}^{2}+f^{2}}-f\right)\\ E_{x-\text{LP}}=a_{1}\;\exp\left(i\varphi_{x1}\right)+a_{2}\;\exp\left(i\varphi_{x2}\right)+a_{3}\;\exp\left(i\varphi_{x3}\right)\\ \varphi_{x-\text{LP}}=abs\left(E_{x-\text{LP}}\right)\times angle\left(E_{x-\text{LP}}\right) \end{array}$$

(Equation 12) $$\begin{array}{l} \varphi_{y1}=-\frac{2\pi }{\lambda }\left(\sqrt{x^{2}+{\left(y-1,400\right)}^{2}+f^{2}}-f\right)\\ \varphi_{y2}=-\frac{2\pi }{\lambda }\left(\sqrt{{\left(x-987\right)}^{2}+{\left(y+987\right)}^{2}+f^{2}}-f\right)\\ \varphi_{y3}=-\frac{2\pi }{\lambda }\left(\sqrt{x^{2}+{\left(y+1,400\right)}^{2}+f^{2}}-f\right)+\pi \\ E_{y-\text{LP}}=b_{1}\;\exp\left(i\varphi_{y1}\right)+b_{2}\;\exp\left(i\varphi_{y2}\right)+b_{3}\;\exp\left(i\varphi_{y3}\right)\\ \varphi_{y-\text{LP}}=abs\left(E_{y-\text{LP}}\right)\times angle\left(E_{y-\text{LP}}\right) \end{array}$$

Here, the amplitude ratio of three foci of the generated *x*-LP component is *a*_1_: *a*_2_: *a*_3_ = 1:2:1, and the same as *y*-LP component (*b*_1_: *b*_2_: *b*_3_ = 1:2:1). In addition, *φ*_*x*-LP_ and *φ*_*y*-LP_ are the phase profiles of the *x-*LP and *y-*LP components of the output wave.

**Figure 7:**
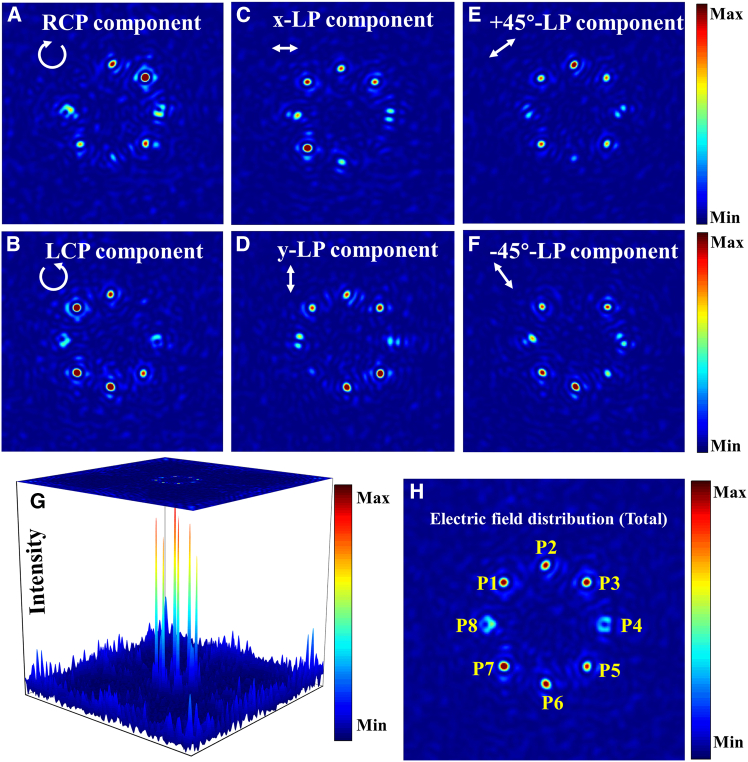
Electric field distributions of polarization components in the focal plane under LCP illumination (A) RCP. (B) LCP. (C) *x*-LP. (D) *y*-LP. (E) +45°-LP. (F) −45°-LP. (G) Intensity of each polarization components in the focal plane. (H) Total electric field distribution in the focal plane.

The all-silicon terahertz metasurface (denoted as 8P1P-MS) was modeled and simulated in CST Microwave Studio’s time-domain solver. The structure, which comprises a 100 × 100 array of meta-atoms, was simulated with open boundary conditions in all three directions. A terahertz plane wave excitation was incident from the -*z*-direction. The numerical simulation results are presented in Figure 7. Figures 7A–7F systematically present the electric field distribution of RCP, LCP, *x*-LP, *y*-LP, +45°-LP, and −45°-LP components on the focal plane, respectively. Then, the spatial focal points are denoted as P1–P8, respectively. As demonstrated in Figures 7A and 7B, focal point P1 is LCP while P3 is RCP. Figures 7C and 7D reveal that focal point P5 corresponds to *y*-LP and P7 corresponds to *x*-LP. Notably, Figures 7C and 7D also reveal that focal points P4 and P8 represent APVB and RPVB, respectively. The ±45°-LP states are localized at focal points P2 (+45°-LP) and P6 (−45°-LP) in Figures 7E and 7F. High polarization purity was achieved at each focus, with low inter-channel crosstalk (see Section S3 in the supplemental information for details). Figure 7G presents the intensity of each focal point across the focal plane, while Figure 7H depicts the total electric field distribution in the focal plane of 8P1P-MS.

### Generation of multi-polarized vortex beams along the longitudinal direction

**Figure 8:**
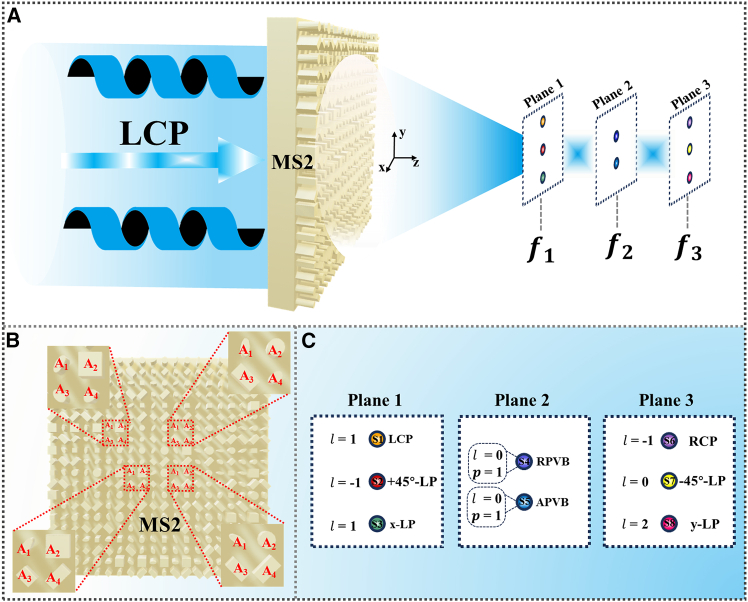
The second designed metasurface 8P3P-MS (A) Schematic of the generation of multi-polarized vortex beams along the longitudinal direction under LCP illumination. (B) Metasurface 8P3P-MS. (C) Pre-designed multi-polarization states can be generated in different focal planes.

Subsequently, we designed a second metasurface capable of generating vector vortex beams with eight polarization states on the three focal planes (8P3P-MS) along the longitudinal direction under LCP illumination, as illustrated in Figure 8. Figure 8B presents the top view of 8P3P-MS. By spatially coupling the four polarization states (RCP, LCP, *x*-LP, and *y*-LP) in the transmitted wave to different focal points, introducing topological charge *l*-encoded in phase modulation and regulating energy weights across each polarization state via amplitude encoding techniques, balanced energy distribution among polarization states in interference superposition points was achieved. This approach enables generating multi-polarization vector vortex beams in different focal planes, with the anticipated polarization state distributions depicted in Figure 8C.

In the design of 8P3P-MS, amplitude encoding is introduced to focus the transmitted waves in accordance with a specified energy distribution. Based on the method of complex amplitude modulation, we need to encode the complex amplitude information into LCP, RCP, *x*-LP, and *y*-LP components, which can be described as Equations 13, 14, 15, and 16:

(Equation 13) $$\begin{array}{l} \varphi_{LL1}=-\frac{2\pi }{\lambda }\left(\sqrt{x^{2}+{\left(y+1,500\right)}^{2}+{f_{1}}^{2}}-f_{1}\right)+l_{1}*\theta \\ \varphi_{LL2}=-\frac{2\pi }{\lambda }\left(\sqrt{x^{2}+{\left(y+750\right)}^{2}+{f_{2}}^{2}}-f_{2}\right)+l_{1}*\theta \\ \varphi_{LL3}=-\frac{2\pi }{\lambda }\left(\sqrt{x^{2}+{\left(y-750\right)}^{2}+{f_{2}}^{2}}-f_{2}\right)+l_{1}*\theta +\pi \\ E_{LL}=m_{1}\;\exp\left(i\varphi_{LL1}\right)+m_{2}\;\exp\left(i\varphi_{LL2}\right)+m_{3}\;\exp\left(i\varphi_{LL3}\right)\\ \varphi_{LL}=abs\left(E_{LL}\right)\times angle\left(E_{LL}\right) \end{array}$$

(Equation 14) $$\begin{array}{l} \varphi_{RR1}=-\frac{2\pi }{\lambda }\left(\sqrt{x^{2}+{\left(y+1,500\right)}^{2}+{f_{3}}^{2}}-f_{3}\right)+l_{2}*\theta \\ \varphi_{RR2}=-\frac{2\pi }{\lambda }\left(\sqrt{x^{2}+{\left(y+750\right)}^{2}+{f_{2}}^{2}}-f_{2}\right)+l_{2}*\theta \\ \varphi_{RR3}=-\frac{2\pi }{\lambda }\left(\sqrt{x^{2}+{\left(y-750\right)}^{2}+{f_{2}}^{2}}-f_{2}\right)+l_{2}*\theta \\ E_{RR}=n_{1}\;\exp\left(i\varphi_{RR1}\right)+n_{2}\;\exp\left(i\varphi_{RR2}\right)+n_{3}\;\exp\left(i\varphi_{RR3}\right)\\ \varphi_{RR}=abs\left(E_{RR}\right)\times angle\left(E_{RR}\right) \end{array}$$

(Equation 15) $$\begin{array}{l} \varphi_{xx1}=-\frac{2\pi }{\lambda }\left(\sqrt{x^{2}+y^{2}+{f_{1}}^{2}}-f_{1}\right)+l_{2}*\theta \\ \varphi_{xx2}=-\frac{2\pi }{\lambda }\left(\sqrt{x^{2}+y^{2}+{f_{3}}^{2}}-f_{3}\right)\\ \varphi_{xx3}=-\frac{2\pi }{\lambda }\left(\sqrt{x^{2}+{\left(y-1,500\right)}^{2}+{f_{1}}^{2}}-f_{1}\right)+l_{1}*\theta \\ E_{xx}=p_{1}\;\exp\left(i\varphi_{xx1}\right)+p_{2}\;\exp\left(i\varphi_{xx2}\right)+p_{3}\;\exp\left(i\varphi_{xx3}\right)\\ \varphi_{xx}=abs\left(E_{xx}\right)\times angle\left(E_{xx}\right) \end{array}$$

(Equation 16) $$\begin{array}{l} \varphi_{yy1}=-\frac{2\pi }{\lambda }\left(\sqrt{x^{2}+y^{2}+{f_{1}}^{2}}-f_{1}\right)+l_{2}*\theta \\ \varphi_{yy2}=-\frac{2\pi }{\lambda }\left(\sqrt{x^{2}+y^{2}+{f_{3}}^{2}}-f_{3}\right)+\pi \\ \varphi_{yy3}=-\frac{2\pi }{\lambda }\left(\sqrt{x^{2}+{\left(y-1,500\right)}^{2}+{f_{3}}^{2}}-f_{3}\right)+l_{3}*\theta \\ E_{yy}=q_{1}\;\exp\left(i\varphi_{yy1}\right)+q_{2}\;\exp\left(i\varphi_{yy2}\right)+q_{3}\;\exp\left(i\varphi_{yy3}\right)\\ \varphi_{yy}=abs\left(E_{yy}\right)\times angle\left(E_{yy}\right) \end{array}$$

Here, the focal lengths *f*_1_, *f*_2_, and *f*_3_ are set to 4,000 μm, 7,000 μm, and 10,000 μm, respectively. Under LCP illumination, the three focal amplitude ratios of the generated LCP component are *m*_1_: *m*_2_: *m*_3_ = 2:1:1, while those of the generated RCP component follow *n*_1_: *n*_2_: *n*_3_ = 2:1:1. For the generated *x*-LP and *y*-LP components, the amplitude ratios are configured as *p*_1_:*p*_2_:*p*_3_ = 1:1:2 and *q*_1_:*q*_2_:*q*_3_ = 1:1:2, respectively. The *l* represents the number of topological charges carried by each polarization state component, governing the orbital angular momentum (OAM) of the vortex beams, and *l*_1_ = 1, *l*_2_ = −1, *l*_3_ = −2.

**Figure 9:**
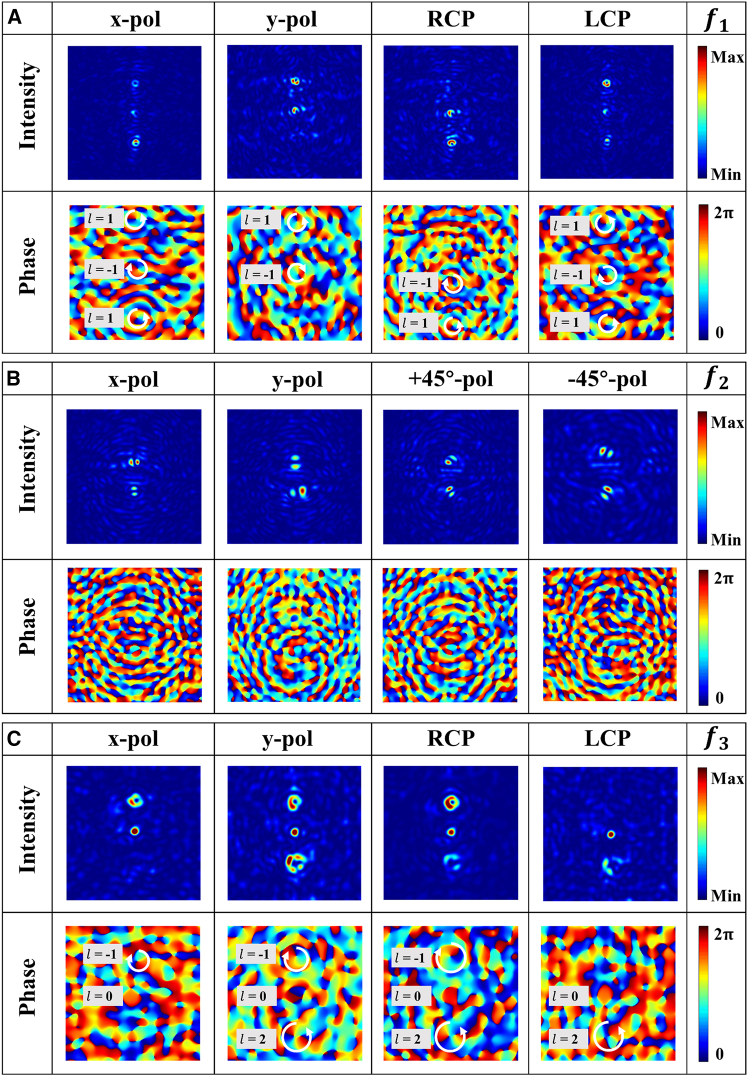
The electric field distribution of polarization state components in different focal planes for 8P3P-MS under LCP illumination (A) Focal plane *f*_1_. (B) Focal. plane *f*_2_. (C) Focal plane *f*_3_.

The spatial focal points are labeled as S1–S8, respectively. Figures 9A and 9C display the electric field intensity distributions and corresponding phase profiles of the four polarization components (*x*-LP, *y*-LP, RCP, and LCP) in the focal planes of *f*_1_ = 4,000 μm and *f*_3_ = 10,000 μm, respectively. The analysis of Figure 9A reveals that the spatial points S3 and S1 correspond to *x*-LP and LCP beams, respectively. From Figure 9C, the spatial points S8 and S7 are identified as *y*-LP and RCP beams, respectively.

**Figure 10:**
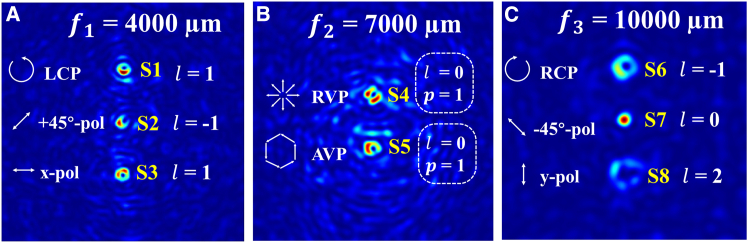
Electric field distribution of 8P3P-MS under LCP illumination at different focal planes (A) Focal plane *f*_1_. (B) Focal plane *f*_2_. (C) Focal plane *f*_3_.

Figure 9B illustrates the electric field intensity distributions and phase profiles of four polarization components (*x*-LP, *y*-LP, +45°-LP, and −45°-LP) at the *f*_2_ = 7,000 μm focal plane. Based on Figure 9B, the spatial points S4 and S5 are RPVB and APVB, respectively. Figure 10 displays the total electric field distribution on the three focal planes. By superposition of polarization states combined with comprehensive analysis of polarization order and topological charge, the space points S1, S2, and S3 are LCP, +45°-LP, and *x*-LP vortex beams carrying topological charges of 1, −1, and 1, respectively. The space points S4 and S5 are RPVB and APVB with topological charge of 0 and polarization order of 1, respectively. The space points S6 and S8 in space are RCP and *y*-LP vortex beams carrying topological charges −1 and 2, respectively. Finally, S7 is a −45°-LP focused beam.

### Meta-atoms verification and Stokes parameters

Figure S1 demonstrates the verification of wavefront modulation using meta-atoms. The spatially average normalized Stokes parameters (*S*_1_, *S*_2_, *S*_3_) for each focal region are summarized in Table S4. Figure S2 displays the degree of polarization (DoP) using bar charts to facilitate intuitive visualization. Figure S3 illustrates the spatially variant polarization states of radially and azimuthally polarized vector beams. Figure S4 illustrates the near-field scanning terahertz microscope employed in experimental characterization to measure the transmitted field’s amplitude, phase, and polarization distributions and verify the topological charges of different polarization states.

### Conclusion

This study presented a spatially interleaved metasurface design strategy that leverages the synergistic modulation of geometric phase and propagation phase to achieve multi-polarization beam generation in the terahertz regime. Our future work will focus on the fabrication and experimental characterization of the proposed devices. The proposed strategy not only enables the creation of complex multi-polarization wavefronts in a 2D plane but also extends this capability to the longitudinal dimension, producing vortex beams with distinct polarization states and topological charges through precise control of polarization components. To validate this multi-polarization generation scheme, two metasurfaces were designed and demonstrated. The first metasurface generates spatially separated focused spots with different polarization states in the focal plane under LCP illumination. The second metasurface produces vortex beams with varying polarization states and topological charges under the same LCP illumination. This work provides a solution for advancing polarization multiplexing. Furthermore, it opens new possibilities for multi-dimensional optical field manipulation, with promising applications in secure information transmission and next-generation terahertz technologies.

### Limitations of the study

Although this study presents progress in multi-polarization beam generation based on metasurfaces, several limitations should be acknowledged. First, the metasurface design demonstrates performance only at specific frequencies, which may limit the generalizability of the results. Second, more complex jamming scenarios were not thoroughly investigated, and these factors may influence the overall performance. In addition, all experiments were conducted under simulation conditions, and further validation in more diverse or real-world environments is required. Our future work will focus on the fabrication and experimental characterization of the proposed devices (see Section S4 in the supplemental information for plan implementation steps).

## Resource availability

### Lead contact

Further information and requests for resources and reagents should be directed to and will be fulfilled by the lead contact, Bo Dai (daibo@usst.edu.cn).

### Materials availability

This study did not generate new reagents.

### Data and code availability

• Data reported in this paper will be shared by the lead contact upon request.
• This paper does not report original codes.
• Any additional information required to reanalyze the data reported in this paper is available from the lead contact upon request.

## Acknowledgments

This work was supported by the 10.13039/501100001809National Natural Science Foundation of China under grants 62205017 and 62475157 and by the 10.13039/501100003399Science and Technology Commission of Shanghai Municipality under grant 24511106500.

## Author contributions

S.H., conceptualization, funding acquisition, supervision, resources, writing – review and editing; Y.L., conceptualization, methodology, validation, simulation, software, original draft; T.T., calculation, visualization writing – review and editing; L.W., investigation, writing – review and editing; B.D., investigation, writing – review and editing; S.Z., investigation, writing – review and editing; D.Z., investigation, writing – review and editing.

## Declaration of interests

The authors declare no conflicts of interest.

## STAR★Methods

### Key resources table

**Table:** 

REAGENT or RESOURCE	SOURCE	IDENTIFIER
**Software and algorithms**		

CST	CST China Co.,LTD.	https://www.cst-china.cn
MATLAB	MathWorks Co.,LTD.	https://www.mathworks.com/products/matlab.html

### Experimental model and study participant details

The CST Microwave Studio software has been employed to analyze the far-field patterns of the proposed metasurfaces. In these numerical simulations, the propagation direction of incident wave is set to be perpendicular to the x-y plane where the metasurface.

### Method details

The simulation is conducted with the CST Microwave Studio software. The boundary conditions in the x and y directions are open, and the boundary conditions in the z direction are open (add space). The designed two metasurfaces consist of a 100∗100 array of unit cells. The material used in the all-dielectric metasurfaces was high resistivity silicon, the dielectric constant was 11.9.

### Quantitation and statistical analysis

The simulation data is produced by CST Microwave Studio software. Figures shown in the main text were produced by Origin and Microsoft PowerPoint from the raw data.

### Additional resources

Any additional information about the simulation and data reported in this paper is available from the lead contact on request.

## Supplemental information

Document S1. Figures S1–S4, Tables S1–S4, and Section S1–S4
