# Supplementary material for: All-silicon terahertz metasurfaces for multi-focus multiplexed polarization generation
Source: iScience. 2025 Dec 16;29(1):114458. doi: 10.1016/j.isci.2025.114458 (PMC12805370; doi:10.1016/j.isci.2025.114458)
Supplement: Document S1. Figures S1–S4, Tables S1–S4, and Section S1–S4 [file mmc1.pdf]

iScience, Volume *29*

Supplemental information

All-silicon terahertz metasurfaces for multi-focus multiplexed polarization generation

Susu Hu, Yongzheng Lu, Tianchen Tang, Li Wei, Bo Dai, Songlin Zhuang, and Dawei Zhang

Supplemental information

S1: Scheme of multiple polarization states generation

Here, to analyze the physical mechanism of complex multiple polarized states producing, we used the Jones matrix model. The Jones matrix representing the linearly polarized transmission wave of a meta-atom can be expressed as $T=\begin{bmatrix} t_{xx} & 0 \\ 0 & t_{yy} \end{bmatrix}$, where $t_{xx}=T_{xx}e^{i\varphi_{xx}}$ and $t_{yy}=T_{yy}e^{i\varphi_{yy}}$.

Herein, t_{xx} and t_{yy} denote the transmission coefficients of the polarization component along the *x* and *y* axis, T_{xx} (T_{yy}) and $\varphi_{xx}(\varphi_{yy})$ are the amplitude and phase under x and y linear polarized (x-LP and y-LP) illumination. Assuming the transmission amplitudes of anisotropic meta-atoms are T_{xx} = T_{yy} = 1, and the rotation angle is θ, then the Jones matrix of the metasurfaces can be expressed as

$$J(\theta)=\mathrm{M}(\theta)\times T\times \mathrm{M}^{-1}(\theta) \qquad \text{(Equation S1)}$$

where $\mathrm{M}(\theta)=\begin{bmatrix} \cos\theta & -\sin\theta \\ \sin\theta & \cos\theta \end{bmatrix}$ is the rotation matrix. Then, $J(\theta)$ can be finally denoted as

$$J(\theta)=\begin{bmatrix} \cos\theta & -\sin\theta \\ \sin\theta & \cos\theta \end{bmatrix}\times\begin{bmatrix} e^{i\varphi_{xx}} & 0 \\ 0 & e^{i\varphi_{yy}} \end{bmatrix}\times\begin{bmatrix} \cos\theta & \sin\theta \\ -\sin\theta & \cos\theta \end{bmatrix} \qquad \text{(Equation S2)}$$

In this article, we define left-handed circular polarization as $|L\rangle=\begin{bmatrix} 1 \\ -i \end{bmatrix}$ and right-handed circular polarization (RCP) as $|R\rangle=\begin{bmatrix} 1 \\ i \end{bmatrix}$. Figure 2 shows the meta-molecule constituting the metasurface, which comprises four types of meta-atoms with distinct physical properties. Meta-atom A_1 functions as a half-wave plate (HWP) with a phase delay of $\Delta\varphi=\varphi_{xx}-\varphi_{yy}=\pi$ between its fast and slow axes. When the rotation angle is 0°, its corresponding Jones matrix can be expressed as

$$J_{A_1}=\begin{bmatrix} 1 & 0 \\ 0 & 1 \end{bmatrix}\times\begin{bmatrix} e^{i\varphi_{xx}} & 0 \\ 0 & e^{i(\varphi_{xx}-\pi)} \end{bmatrix}\times\begin{bmatrix} 1 & 0 \\ 0 & 1 \end{bmatrix}=\begin{bmatrix} e^{i\varphi_{xx}} & 0 \\ 0 & -e^{i\varphi_{xx}} \end{bmatrix} \qquad \text{(Equation S3)}$$

For LCP incident light with $E_{in}=\begin{bmatrix} 1 \\ -i \end{bmatrix}$, the output light is

$$E_{out1}=J_{A_1}\times E_{in}=\begin{bmatrix} e^{i\varphi_{xx}} & 0 \\ 0 & -e^{i\varphi_{xx}} \end{bmatrix}\times\begin{bmatrix} 1 \\ -i \end{bmatrix}=e^{i\varphi_{xx}}\begin{bmatrix} 1 \\ i \end{bmatrix} \qquad \text{(Equation S4)}$$

According to equation S4, A_1 converts LCP light into the RCP component of the transmitted wave. Meta-atom A_2, an isotropic meta-atom, exhibits a phase retardation of $\Delta\varphi=\varphi_{xx}-\varphi_{yy}=0$ between its fast and slow axes. When the rotation angle is 0°, its corresponding Jones matrix is expressed as

$$J_{A_2}=\begin{bmatrix} 1 & 0 \\ 0 & 1 \end{bmatrix}\times\begin{bmatrix} e^{i\varphi_{xx}} & 0 \\ 0 & e^{i(\varphi_{xx}-0)} \end{bmatrix}\times\begin{bmatrix} 1 & 0 \\ 0 & 1 \end{bmatrix}=\begin{bmatrix} e^{i\varphi_{xx}} & 0 \\ 0 & e^{i\varphi_{xx}} \end{bmatrix} \qquad \text{(Equation S5)}$$

resulting in an output light as

$$E_{out2}=J_{A_2}\times E_{in}=\begin{bmatrix} e^{i\varphi_{xx}} & 0 \\ 0 & e^{i\varphi_{xx}} \end{bmatrix}\times\begin{bmatrix} 1 \\ -i \end{bmatrix}=e^{i\varphi_{xx}}\begin{bmatrix} 1 \\ -i \end{bmatrix} \qquad \text{(Equation S6)}$$

According to equation S6, element A_2 can maintain the LCP polarization property unchanged. Meta-atoms A_3 and A_4 exhibit the characteristics of quarter-wave plates, with phase retardation between their fast and slow axes being $\Delta\varphi=\varphi_{xx}-\varphi_{yy}=\pi/2$. When A_3 and A_4 are rotated by angles of $\pi/4$ and $-\pi/4$, respectively, it follows that

$$J_{A_3}=\begin{bmatrix} \frac{\sqrt{2}}{2} & -\frac{\sqrt{2}}{2} \\ \frac{\sqrt{2}}{2} & \frac{\sqrt{2}}{2} \end{bmatrix}\times\begin{bmatrix} e^{i\varphi_{xx}} & 0 \\ 0 & e^{i\varphi_{yy}} \end{bmatrix}\times\begin{bmatrix} \frac{\sqrt{2}}{2} & \frac{\sqrt{2}}{2} \\ -\frac{\sqrt{2}}{2} & \frac{\sqrt{2}}{2} \end{bmatrix}=\frac{\sqrt{2}}{2}e^{i\varphi_{xx}}\begin{bmatrix} e^{i\frac{\pi}{4}} & e^{-i\frac{\pi}{4}} \\ e^{-i\frac{\pi}{4}} & e^{i\frac{\pi}{4}} \end{bmatrix} \qquad \text{(Equation S7)}$$

$$E_{out3} = J_{A_3} \times E_{in} = \frac{\sqrt{2}}{2} e^{i\varphi_{xx}} \begin{bmatrix} e^{i\frac{\pi}{4}} & e^{-i\frac{\pi}{4}} \\ e^{-i\frac{\pi}{4}} & e^{i\frac{\pi}{4}} \end{bmatrix} \times \begin{bmatrix} 1 \\ -i \end{bmatrix} = \sqrt{2} e^{i(\varphi_{xx} - \frac{\pi}{4})} \begin{bmatrix} 0 \\ 1 \end{bmatrix} \quad \text{(Equation S8)}$$

$$J_{A_4} = \begin{bmatrix} \frac{\sqrt{2}}{2} & \frac{\sqrt{2}}{2} \\ -\frac{\sqrt{2}}{2} & \frac{\sqrt{2}}{2} \end{bmatrix} \times \begin{bmatrix} e^{i\varphi_{xx}} & 0 \\ 0 & e^{i\varphi_{yy}} \end{bmatrix} \times \begin{bmatrix} \frac{\sqrt{2}}{2} & -\frac{\sqrt{2}}{2} \\ \frac{\sqrt{2}}{2} & \frac{\sqrt{2}}{2} \end{bmatrix} = \frac{\sqrt{2}}{2} e^{i\varphi_{xx}} \begin{bmatrix} e^{i\frac{\pi}{4}} & -e^{-i\frac{\pi}{4}} \\ -e^{-i\frac{\pi}{4}} & e^{i\frac{\pi}{4}} \end{bmatrix} \quad \text{(Equation S9)}$$

$$E_{out4} = J_{A_4} \times E_{in} = \frac{\sqrt{2}}{2} e^{i\varphi_{xx}} \begin{bmatrix} e^{i\frac{\pi}{4}} & -e^{-i\frac{\pi}{4}} \\ -e^{-i\frac{\pi}{4}} & e^{i\frac{\pi}{4}} \end{bmatrix} \times \begin{bmatrix} 1 \\ -i \end{bmatrix} = \sqrt{2} e^{i(\varphi_{xx} + \frac{\pi}{4})} \begin{bmatrix} 1 \\ 0 \end{bmatrix} \quad \text{(Equation S10)}$$

According to equations S8 - S10, A_3 and A_4 can convert incident LCP beams into y-LP and x-LP components, respectively. Then, the secondary energy redistribution among the four polarization states (RCP, LCP, y-LP, and x-LP) in transmitted waves enables superposition of spatially diverse polarization states to generate multiple derived polarization modes, as illustrated in Figure 2. The specific analysis proceeds as follows:

$$\frac{1}{2} e^{ip\varphi} \begin{bmatrix} 1 \\ -i \end{bmatrix} + \frac{1}{2} e^{-ip\varphi} \begin{bmatrix} 1 \\ i \end{bmatrix} = e^{i\varphi} \begin{bmatrix} \cos p\varphi \\ \sin p\varphi \end{bmatrix} \quad \text{(Equation S11)}$$

$$\frac{1}{2} e^{ip\varphi} \begin{bmatrix} 1 \\ -i \end{bmatrix} + \frac{1}{2} e^{-ip\varphi} \times e^{i\pi} \begin{bmatrix} 1 \\ i \end{bmatrix} = i \begin{bmatrix} -\sin p\varphi \\ \cos p\varphi \end{bmatrix} \quad \text{(Equation S12)}$$

Equation S11 demonstrates that a LCP vortex beam with an angular quantum number *p* and identical initial phase, when combined with a RCP vortex beam of angular quantum number -*p*, can be coherently synthesized into a radial VB with a polarization order of *p*. Introducing a phase delay of π to the RCP vortex beam transforms the synthesized beam into an azimuthal VB, as shown in equation S12. Consequently, superposition of RCP and LCP waves with equal amplitudes and identical or opposite initial phases generates RPVBs or APVBs.

For a +45° linearly polarized beam, the corresponding Jones vector can be considered as the superposition of x-LP and y-LP components with identical amplitudes and initial phases, as expressed in equation S13. Similarly, a -45°-LP beam corresponds to the superposition of x-LP and y-LP components with equal amplitudes but a phase difference of π, as formulated in equation S14.

$$\begin{bmatrix} 1 \\ 0 \end{bmatrix}_{x-lp} + \begin{bmatrix} 0 \\ 1 \end{bmatrix}_{y-lp} = \begin{bmatrix} 1 \\ 1 \end{bmatrix}_{+45°\text{-}lp} \quad \text{(Equation S13)}$$

$$\begin{bmatrix} 1 \\ 0 \end{bmatrix}_{x-lp} - \begin{bmatrix} 0 \\ 1 \end{bmatrix}_{y-lp} = \begin{bmatrix} 1 \\ -1 \end{bmatrix}_{-45°\text{-}lp} \quad \text{(Equation S14)}$$

S2: Meta-atom design and verification

In metasurfaces design, each meta-atom corresponds to one polarization state and one independent phase distribution. At one working wavelength, achieving a full 0-2π phase coverage using standalone elliptical or rectangular pillars as meta-atoms presents significant challenges. Even if meta-atoms theoretically capable of spanning the entire phase range could be identified, their transmission efficiency often remains suboptimal. To address this dual limitation of phase coverage and high efficiency, this work proposes a hybrid architecture that strategically integrates both elliptical and rectangular pillars in the meta-atom arrangement.

Tables S1, S2, and S3 summarize the geometric parameters, transmission coefficients (T_{xx}, T_{yy}), propagation phases (Ph_{xx}, Ph_{yy}), and phase retardation ($\Delta Ph = | Ph_{xx} - Ph_{yy} |$) for the three selected types of meta-atoms. The geometric parameters along the *x*- and *y*-axes are denoted

as *w* and *l*, respectively, with subscript *e* indicating elliptical nanopillars and subscript *r* representing rectangular nanopillars. As shown in Table S1, the meta-atoms exhibit phase retardation $\Delta Ph \approx \pi$, functioning as half-wave plates. Table S2 demonstrates that the six selected meta-atoms A_2 achieve full 0–2π coverage for both Ph_{xx} and Ph_{yy} with incremental steps of π/3, while maintaining a relatively high smooth transmission amplitudes (~0.76). The phase retardation is $\Delta Ph \approx 0$, indicating that A_2 can play the function of full wave plate. Table S3 reveals that the selected six meta-atoms of A_3 complete 0–2π phase coverage with π/3 increments, accompanied by stable transmission amplitudes (~0.8). The consistent phase retardation $\Delta\varphi \approx \pi/2$ establishes their functionality as quarter-wave plates.

Table S1. Optical Properties and Geometric Parameters of Type A_1 Meta-Atoms, related to STAR Methods.

	x-axis parameter (μm)	*y-axis* parameter (μm)	T_{xx}	T_{yy}	Ph_{xx}	Ph_{yy}	$\|\Delta Ph\|$
A_1	w_e = 21	l_e = 45	0.77	0.78	136.92	-43.08	180.00

Table S2. Optical Properties and Geometric Parameters of Type A_2 Meta-Atoms, related to STAR Methods.

	x-axis parameter (μm)	*y-axis* parameter (μm)	T_{xx}	T_{yy}	Ph_{xx}	Ph_{yy}	$\|\Delta Ph\|$
A_{21}	w_e = 56	l_e = 56	0.68	0.66	-222.83	-222.84	0.01
A_{22}	w_e = 43	l_e = 44	0.71	0.69	-165.47	-165.66	0.19
A_{23}	w_e = 38	l_e = 38	0.81	0.81	-105.88	-105.92	0.04
A_{24}	w_r = 31	l_r = 31	0.76	0.76	-43.19	-43.19	0.00
A_{25}	w_r = 29	l_r = 29	0.72	0.72	16.03	16.03	0.00
A_{26}	w_r = 57	l_r = 57	0.73	0.79	78.15	78.16	0.01

Table S3. Optical Properties and Geometric Parameters of Type A_3 Meta-Atoms, related to STAR Methods.

	x-axis parameter (μm)	*y-axis* parameter (μm)	T_{xx}	T_{yy}	Ph_{xx}	Ph_{yy}	$\|\Delta Ph\|$
A_{31}	w_r = 34	l_r = 65	0.77	0.75	-178.84	-266.47	87.63
A_{32}	w_r = 30	l_r = 57	0.74	0.76	-119.40	-210.14	90.74
A_{33}	w_r = 29	l_r = 42	0.79	0.75	-59.77	-149.54	89.77
A_{34}	w_r = 28	l_r = 35	0.73	0.73	-1.83	-87.17	89
A_{35}	w_e = 29	l_e = 37	0.74	0.74	60.95	-32.95	93.9
A_{36}	w_e = 25	l_e = 36	0.79	0.74	120.79	26.11	94.68

Before designing functional metasurfaces, it is essential to validate whether the selected meta-atoms meet the design requirements. Based on the chosen meta-atoms, a straightforward demonstration of wavefront manipulation for LCP, RCP, x-linear polarized, and y-linear polarized incidences is subsequently presented. As shown in Figure S1(a), arranging A_1-type meta-atoms with a spatial rotation angle of π/3 successfully achieves the beam deflection phenomenon illustrated in Figure S1(b), preliminarily confirming the rationality of the meta-atoms selection. Numerical simulations further demonstrate that, under LCP and RCP illumination (Figure S1(c)), the RCP and LCP transmitted wave are deflected toward opposite symmetric angles, respectively. Figures S1(d) and (e) display the arrangement of A_2-type meta-atoms under 0–2π phase coverage. Figure S1(f) reveals that under x-LP and y-LP illumination, the equiphase surfaces of the generated x- and y-polarized waves exhibit distinct tilting, indicating successful beam steering. Similarly, Figures S1(g) and (h) present the configuration of A_3-type meta-atoms with 0–2π phase profiles. Figure S1(i) demonstrates that under x- and y-linear polarized illumination, the equiphase surfaces of the corresponding x- and y-polarized waves exhibit the anticipated tilting characteristics as predicted in Figure S1(h), confirming the realization of beam deflection.

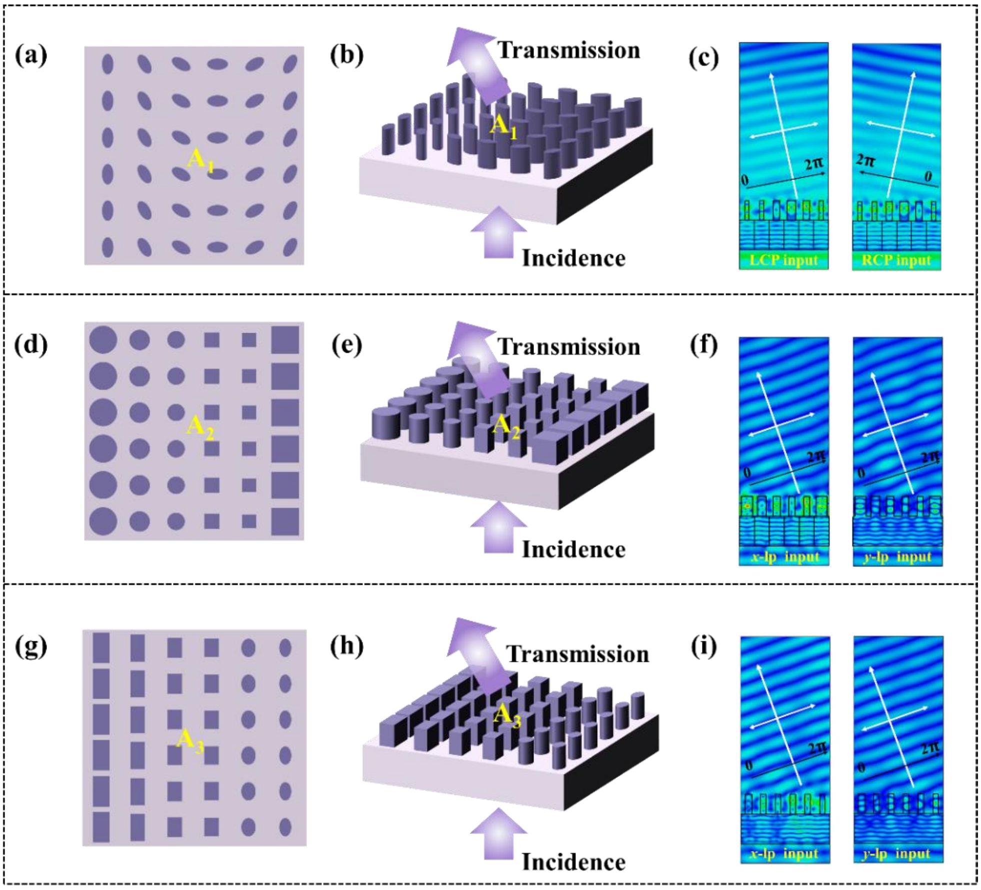

Figure S1. Verification of wavefront modulation using meta-atoms, related to Figure 5.
(a) Schematic of the spatial rotation arrangement of A_1-type meta-atoms (rotational spacing of π/3). (b) Theoretically designed beam deflection and (c) Numerical simulation results under LCP and RCP illumination, demonstrating symmetric angular offsets of the transmitted RCP and LCP components, respectively. (d) Arrangement configuration of A_2-type meta-atoms under a 0–2π linear phase gradient. (e) Theoretically designed beam deflection based on (d). (f) The corresponding tilting characteristics of equiphase surfaces for orthogonal x- and y-lp components under illumination. (g) Arrangement configuration of A_3-type meta-atoms under a 0–2π linear phase gradient. (h) Theoretically designed beam deflection based on (g) and (i) its wavefront modulation characteristic

S3: Results and analysis

Quantitative analysis of polarization purity and inter-channel crosstalk is essential, and we have conducted a comprehensive Stokes parameter analysis for multiple focal spots. In our post-processing of the simulation data, we extracted the optical intensities ($I_{\text{x-LP}}$, $I_{\text{y-LP}}$, $I_{\text{+45-LP}}$, $I_{\text{-45-LP}}$, I_{RCP}, I_{LCP}) for each focal region under six different polarization bases. Based on the standard definition of the Stokes parameters:

$$S_1 = I_{\text{x-LP}} - I_{\text{y-LP}}$$
$$S_2 = I_{\text{+45-LP}} - I_{\text{-45-LP}} \qquad \text{(Equation S15)}$$
$$S_3 = I_{\text{RCP}} - I_{\text{LCP}}$$

We calculated the average normalized Stokes parameters (S_1, S_2, S_3) for each focal region (see Table S4). The results including degree of polarization (DoP) are summarized in bar charts for clear visualization (see Figure S2). As shown, the designed polarization states exhibit high purity. For instance, the RCP focus (P3) shows S_3 = 0.978, while the +45° linearly polarized focus (P2) achieves S_2 = 0.962. The degree of polarization (DoP) at all focal spots exceeds 0.94. These values quantitatively confirm the high polarization purity achieved at each focus.

Table S4. Stokes parameters and DoP at P1, P2, P3, P5, P6, and P7, related to Figure 7.

	Designed polarization state	S_1	S_2	S_3	DoP
P1	LCP	0.015	0.028	-0.974	0.975
P2	+45°- LP	-0.035	0.962	-0.018	0.963
P3	RCP	-0.022	-0.041	0.978	0.979
P5	y- LP	-0.945,	0.012	0.026	0.945
P6	-45°- LP	0.028	0.955	0.015	0.956
P7	x- LP	0.968	0.021	-0.032	0.969

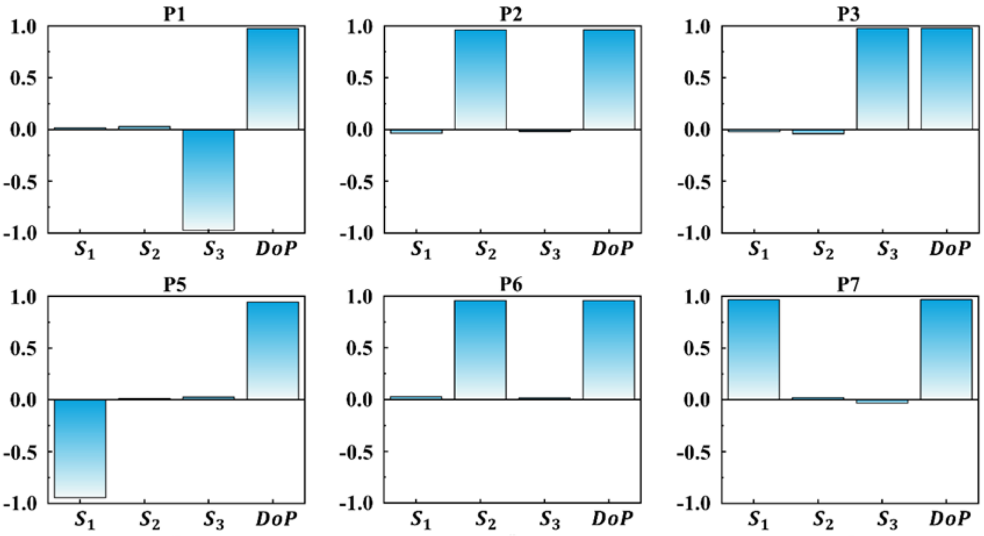

Figure S2. Stokes parameters and degree of polarization (DoP) at focal points P1, P2, P3, P5, P6, and P7, related to Figure 6 and 7.

For radially and azimuthally polarized vector beams, their spatially variant polarization states cannot be fully described by Stokes parameters at a single point. As shown in Figure S3 (a), the x- and y-components of the P4 and P8 foci after passing through an analyzer are displayed, while (b) and (c) present the polarization distribution vector maps of the focal planes for P4 and P8, respectively.

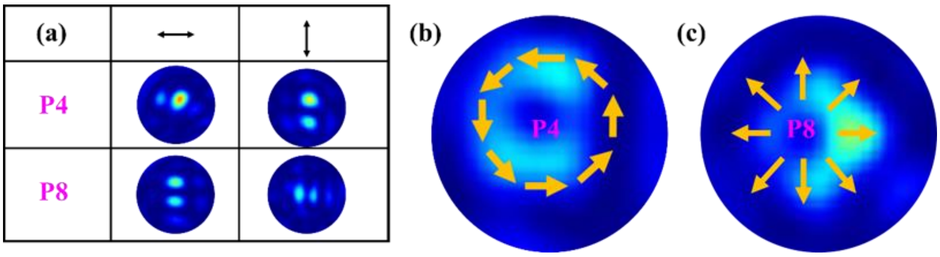

Figure S3. Foci P4 and P8, related to Figure 7.
(a) X- and y-polarized components for foci P4 and P8. Polarization distribution vector maps of the focal planes for (b) P4 and (c) P8.

The above supplementary analysis convincingly validates that our proposed metasurface can generate multiple optical channels with well-defined polarization states and low inter-channel crosstalk.

S4: Future work

Our future work will focus on the fabrication and experimental characterization of the proposed devices. The designed sample can utilize inductively coupled plasma (ICP) etching technology to fabricate. A thin layer of chromium (Cr) is first evaporated onto the silicon substrate to facilitate the adhesion of the photoresist during spin-coating. The photoresist is then spin-

coated onto the chromium layer, and the desired pattern is formed through processes such as masking, exposure, curing, and development. The silicon sample is subsequently etched according to the pattern using SF6 as the etching gas. Finally, the chromium layer and photoresist are removed. The depth of the silicon structure is determined by the etching time, with a relative dimensional error not exceeding ±2%.

For experimental characterization, a near-field scanning terahertz microscope (as shown in Figure S4) can be employed along with specific near-field probing techniques to directly measure the amplitude, phase, and polarization distributions of the transmitted field, thereby verifying the topological charges carried by different polarization states. This platform utilizes a terahertz time-domain spectroscopy system as its core, enabling direct measurement of electric field distribution at the focal plane with nanoscale resolution through scanning with a near-field probe. We anticipate that, based on the current level of micro-nano fabrication technology, the performance of the fabricated devices will closely match our simulation results.

In addition, the results in Figure 7 and Figure 9 are noisy and the quality of the vortex beam is not very high compared with the experimental results in the literatures. We plan to adopt a deep-learning-assisted optimization framework for meta-atom design in our future work. By training a neural network to map geometric parameters to electromagnetic responses (phase and amplitude), we can efficiently generate a much larger and more refined meta-atom library. This will enable the selection of superior unit cells for the array and fundamentally enhance the quality of the simulated output.

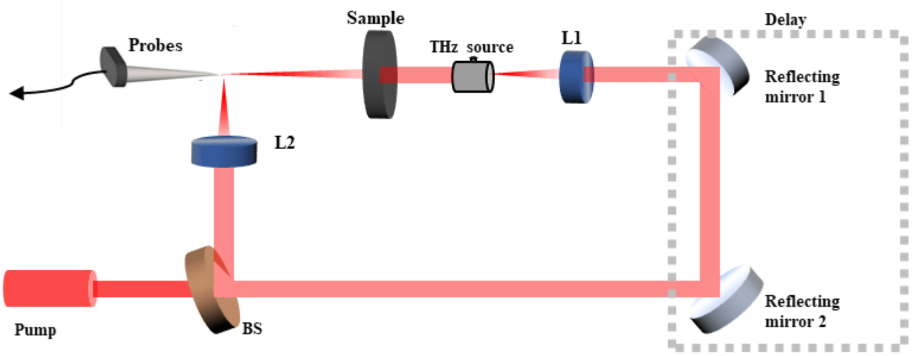

Figure S4. The setup of the near-field scanning terahertz microscopy, related to the STAR Methods.
